# An NmrA-Like Protein, Lws1, Is Important for Pathogenesis in the Woody Plant Pathogen *Lasiodiplodia theobromae*

**DOI:** 10.3390/plants11172197

**Published:** 2022-08-24

**Authors:** Junbo Peng, Janith V. S. Aluthmuhandiram, K. W. Thilini Chethana, Qi Zhang, Qikai Xing, Hui Wang, Mei Liu, Wei Zhang, Xinghong Li, Jiye Yan

**Affiliations:** 1 Beijing Key Laboratory of Environment Friendly Management on Diseases and Pests of North China Fruits, Institute of Plant and Environment Protection, Beijing Academy of Agriculture and Forestry Sciences, Beijing 100097, China; 2 Center of Excellence in Fungal Research, Mae Fah Luang University, Chiang Rai 57100, Thailand; 3 School of Science, Mae Fah Luang University, Chiang Rai 57100, Thailand

**Keywords:** *Lasiodiplodia theobromae*, short-chain dehydrogenase/reductase, pathogenicity, nutrition metabolism

## Abstract

The NmrA-like proteins have been reported to be important nitrogen metabolism regulators and virulence factors in herbaceous plant pathogens. However, their role in the woody plant pathogen *Lasiodiplodia theobromae* is less clear. In the current study, we identified a putative NmrA-like protein, Lws1, in *L. theobromae* and investigated its pathogenic role via gene silencing and overexpression experiments. We also evaluated the effects of external carbon and nitrogen sources on *Lws1* gene expression via qRT-PCR assays. Moreover, we analyzed the molecular interaction between Lws1 and its target protein via the yeast two-hybrid system. The results show that Lws1 contained a canonical glycine-rich motif shared by the short-chain dehydrogenase/reductase (SDR) superfamily proteins and functioned as a negative regulator during disease development. Transcription profiling revealed that the transcription of *Lws1* was affected by external nitrogen and carbon sources. Interaction analyses demonstrated that Lws1 interacted with a putative GATA family transcription factor, LtAreA. In conclusion, these results suggest that Lws1 serves as a critical regulator in nutrition metabolism and disease development during infection.

## 1. Introduction

Filamentous fungi are capable of utilizing a diverse array of compounds as nitrogen sources during their life cycle. Production of the necessary permease and catabolic enzymes, as well as the uptake system required for the utilization of nitrogen resources, is mediated by a general mechanism, known as nitrogen metabolite repression (NMR). In NMR, the expression of many structural genes involved in nitrogen metabolism remains at a low level in the presence of ammonium or glutamine (nitrogen-sufficient conditions), but it is elevated in the presence of poorer nitrogen sources such as alanine (nitrogen-limiting conditions) [1,2,3,4,5]. To date, many of the principal transcription factors involved in the regulation of NMR have been identified and well characterized in various fungi, including *Aspergillus nidulans* [6,7,8], *Neurospora crassa* [7,9], *Penicillium chrysogenum* [10], *Saccharomyces cerevisiae* [11,12,13], *Magnaporthe grisea* [14], *Aspergillus oryzae* [15], *Gibberella fujikuroi* [16], *Aspergillus flavus* [17] (Han et al., 2016), and *Fusarium graminearum* [18].

In the filamentous fungus *A. nidulans*, this phenomenon depends on a positively acting regulator, AreA, which is a GATA family transcription activator with a C_2_C_2_ zinc-finger DNA-binding domain that recognizes the consensus HGATAR motif [2,5,6,7,8]. It has been demonstrated that the expression of this principal regulator is modulated by at least two signaling mechanisms [19]. One mechanism that modulates AreA activity involves accelerated deadenylation and rapid degradation of the *AreA* transcript in the presence of sufficient ammonium or glutamine. This signaling mechanism has been shown to require a region of 218 nucleotides within the 3′ untranslated region (UTR) of the *AreA* transcript [19,20,21]. Under nitrogen-limiting conditions, *AreA* levels are upregulated by autogenous transcriptional control via the GATAR sequences in the *AreA* N-terminal region [2,22]. Another mechanism acts post-translationally [20]. It has been proposed that the control of AreA activity involves the protein TamA, which interacts with the C-terminal residues of AreA and functions as a co-activator of AreA in regulating the expression of genes subject to nitrogen metabolite repression [3,23,24]. In addition, the regulation of AreA activity also involves the transcriptional repressor NmrA interacting with the DNA-binding domain and extreme C-terminus of AreA to prevent the activation of nitrogen catabolic genes [2,3,5,23,25,26,27]. The control of AreA activity by NmrA is independent of the regulation of *AreA* mRNA stability, as the partially derepressed phenotypes associated with the C-terminus of the AreA or NmrA protein and the 3′ UTR of *AreA* are additive [2,3,5,19,25].

It has been determined that the crystal structure of NmrA resembles the short-chain dehydrogenase/reductase (SDR) superfamily and comprises an N-terminal Rossmann fold characterized by a canonical structural motif for dinucleotide binding [3,4,27,28]. SDRs are able to metabolize a wide range of substrates, such as alcohols, aldehydes, ketones, enoates, imines, steroids, polycyclic aromatic hydrocarbons, and retinoids [29,30]. Moreover, it has been found that SDR proteins function as dimers or tetramers, and dimerization is a prerequisite for their catalysis [31]. However, previous research has revealed that the NmrA protein has an incomplete active site motif and is monomeric; therefore, it is unlikely to have SDR enzyme activity [27]. Although the molecular functions of the NmrA protein have been well studied, information on its homolog in the opportunistic plant pathogen *L. theobromae* remains quite poor.

The ascomycete fungus *L. theobromae*, a member of the *Botryosphaeriaceae* family, is able to infect a wide range of economically important plants [32,33,34,35] and has become one of the biggest threats to vineyard sustainability worldwide. The vast economic damage caused by this fungus has driven increased efforts to investigate its distribution, lifestyle, and virulence. In nature, the fungus can live latently inside hosts for an extended period of time without observable disease symptoms [35,36,37]. When the environmental conditions are favorable for its infection and colonization [33,36,37], this pathogen can cause serious disease symptoms including internal brown wood streaking, necrotic lesions, discoloration in the outer xylem, perennial cankers, external bud necrosis or death, leaf spots, dead arms, shoot dieback, and bunch rot [38]. During the last decade, an array of studies aiming to reveal the pathogenesis of this fungus have been performed, and some important progress has been made. Multi-omics analyses have identified large numbers of pathogenicity-related factors in *L. theobromae* [33,34,37,39,40,41]. Importantly, several virulence factors, such as LtLysM1 [42], LtEpg1 [43], and LtCFEMs [35], have been cloned and characterized in detail. Moreover, Aluthmuhandiram et al. [44] isolated two phytotoxins, namely, indole-3-carboxylic acid and jasmonic acid, and tested their virulence against grapevine. In the future, more attention should be paid to investigating the colonization process and pathogenicity mechanism if we aspire to defeat this fungus.

In the current study, one NmrA-like protein was identified and characterized in *L. theobromae*. Phenotypic analyses showed that this protein negatively regulated disease development during infection. We proposed that the protein is important for the weak virulence sustainability of *L. theobromae*, and therefore, it was named Lws1. Moreover, Lws1 interacted with a putative GATA transcription factor, LtAreA. Further transcription profiling showed that Lws1 responded to external carbon and nitrogen nutrition. These results suggest that Lws1, together with LtAreA, participates in nutrition metabolism and pathogenesis in *L. theobromae*.

## 2. Results

### 2.1. Structural Features of Lws1 Protein

**Figure 1:**
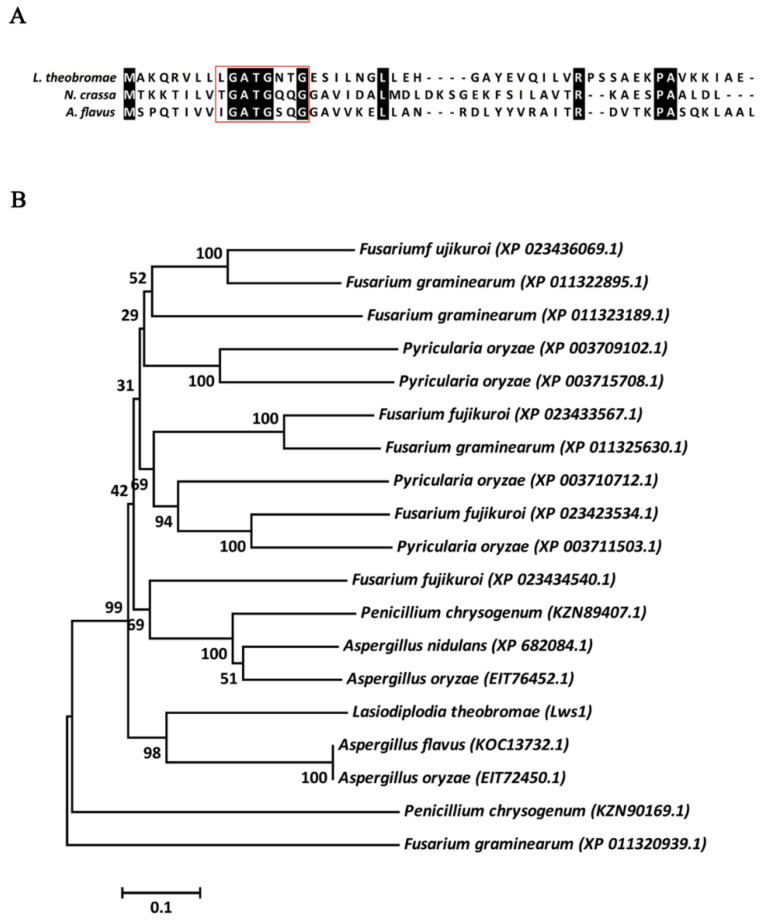
Structural and phylogenetic analyses of NmrA homologs in *L. theobromae* and other filamentous fungi. (**A**) Multiple sequence alignments of NmrA homologs (N-terminal amino acid residues 1–50) from *L. theobromae*, *N. crassa*, and *A. flavus*. Amino acid sequences were aligned using the ClustalX2 program and then edited with Jalview software. The same amino acids are highlighted in black. Residues boxed in red denote the conserved glycine-rich motif of the Rossmann fold. (**B**) Phylogenetic analyses of NmrA homologs from various fungal species. The phylogenetic tree was generated using amino acids sourced from the NCBI database via MEGA7 with the neighbor-joining method, with 2000 replicates. Bootstrap percentage support for each branch is marked at the nodes.

Previous work by our laboratory has identified many genes that have been predicted to participate in nutrition metabolism and pathogenicity in *L. theobromae*. Within the gene lists, we identified a candidate transcription regulator annotated as an NmrA-like protein (hereinafter Lws1, PFAM domain PF05368). Multiple sequence alignments of the N-terminal residues (amino acids 1–50) of the Lws1 protein with its homologs from *N. crassa* and *A. flavus* showed that all three proteins contained a canonical glycine-rich motif (Figure 1A), which was shared by the SDR superfamily proteins.

To analyze the conservatism of NmrA homologs, phylogenetic analyses of NmrA-like proteins from a set of fungi that belong to the Ascomycota phylum were performed. To obtain the sequences, BLASTP searches against the NCBI database were performed using the Lws1 amino acid sequence as the query, with an E-value of 1 × 10^−10^ as the cutoff. In comparison with its homologs, Lws1 is highly conserved across the Ascomycota phylum (Figure 1B), supporting the likelihood of similar functions and structural features of these homologous proteins.

### 2.2. Regulation of the Pathogenicity of L. theobromae by Lws1

**Figure 2:**
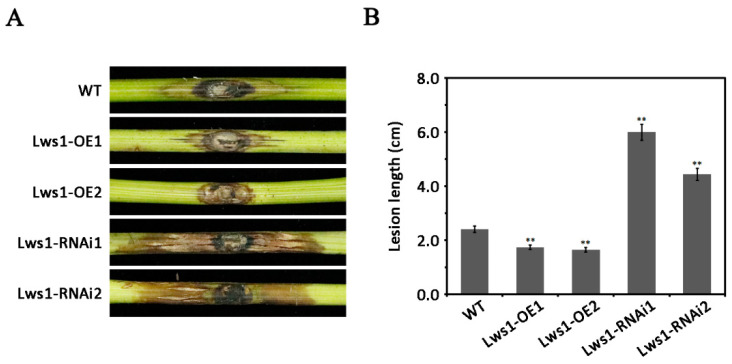
Regulation of Lws1 in the virulence of *L. theobromae*. (**A**) Pathogenicity tests of the wild type (WT), overexpressed transformants (Lws1-OE1 and Lws1-OE2), and silenced transformants (Lws1-RNAi1 and Lws1-RNAi2). Mycelial plugs of these strains (5 mm in diameter) were inoculated on susceptible grapevine green shoots and then placed inside a chamber with constant humidity and temperature. Images were photographed at 3 days post-inoculation (dpi). (**B**) Comparison of the lesion length caused by the strains mentioned in (**A**). The lesion lengths of these strains were quantified at 3 dpi. At least five biological replicates of each strain were tested. A representative set of data is presented. Statistically significant differences are marked by asterisks and were evaluated using one-way analysis of variance (ANOVA) and least significant difference (LSD) tests. ** α = 0.01.

To functionally characterize the *Lws1* gene, we overexpressed and silenced the gene in vivo using the polyethylene glycol (PEG)-mediated transformation method. The wild type, overexpressed transformants of *Lws1* (Lws1-OE1 and Lws1-OE2), and silenced transformants of *Lws1* (Lws1-RNAi1 and Lws1-RNAi2) were inoculated on the detached grapevine shoots. Compared to the wild type, grapevine tissues infected by overexpressed transformants exhibited a marginal reduction in lesion length (Figure 2A,B). The silenced transformants, however, caused a significantly increased lesion length on grapevine shoots (Figure 2A,B), indicating that SDR1 functioned as a negative regulator during disease development.

### 2.3. Interaction between Lws1 and LtAreA

To further explore the molecular mechanism of Lws1, we performed a yeast two-hybrid screening with Lws1 as the bait against the *L. theobromae* cDNA library. Within the potentially interacting targets, one predicted NMR regulator, LtAreA, was selected for subsequent analyses.

**Figure 3:**
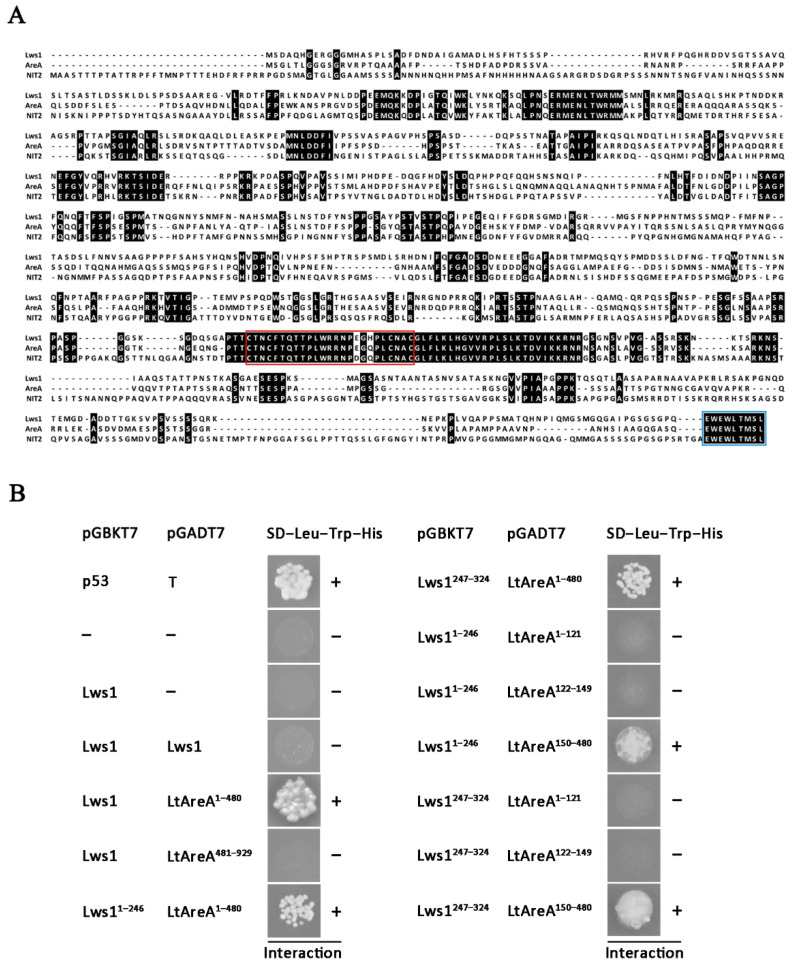
Interaction between Lws1 and LtAreA. (**A**) Multiple sequence alignments of AreA homologs from *L. theobromae*, *A. nidulans*, and *N. crassa*. Residues boxed in red denote the conserved C_2_C_2_ motif. Residues boxed in blue signify the same motif in the extreme C-terminus. (**B**) The *Lws1* cDNA or its truncated forms were cloned into the bait vector *pGBKT7*, and the *LtAreA* cDNA or its truncated forms were cloned into the prey vector *pGADT7*. Yeast cells expressing the prey and bait vectors were tested for their growth on synthetic dropout media (SD−Leu−Trp−His). Yeast transformants expressing the empty prey vector *pGADT7* with the empty bait vector *pGBKT7* or with the Lws1 bait vector (*pGBKT7-Lws1*) were used as negative controls. A yeast transformant expressing the *pGADT7-T* and *pGBKT7-53* vectors was used as a positive control. Lws1^1–246^ signifies an Lws1-truncated fragment that contains amino acids from positions 1 to 246.

LtAreA was predicted to encode a C_2_C_2_ zinc-finger-type GATA domain-binding transcription factor. Sequence alignments showed that LtAreA shared 41.26% and 40.06% amino acid identities with its homologs AreA from *A. nidulans* and NIT2 from *N. crassa*, respectively. Additionally, the zinc-finger DNA-binding motif and the nine C-terminal amino acids were also highly conserved among them (Figure 3A).

To confirm the interaction between LtAreA and Lws1, we performed a yeast two-hybrid analysis in which *Lws1* cDNA was cloned into the bait vector and *LtAreA* cDNA was cloned into the prey vector of the GAL4 two-hybrid system. Using this system, we detected a strong interaction between LtAreA and Lws1 molecules.

To further map the key regions of Lws1 and LtAreA that interacted with each other, we constructed a suite of bait and prey vectors carrying the truncated fragments of Lws1 and LtAreA, respectively, and then simultaneously expressed each bait and prey vector pair in yeast. The obtained results show that Lws1 interacted with LtAreA via its N-terminal moiety (LtAreA^150–480^), rather than its C-terminal moiety (LtAreA^481–929^), indicating that the putative DNA-binding domain of LtAreA (LtAreA^695–745^) is not the key region that physically interacts with Lws1 (Figure 3B).

Usually, SDR proteins function as dimers or tetramers, and the dimerization of SDR proteins is required for their catalysis. Here, we were interested in determining whether Lws1 had the capacity to form multimers based on a protein–protein interaction. Therefore, we constructed the prey vector *pGADT7-Lws1* and bait vector *pGBKT7-Lws1* and transformed both vectors into yeast AH109 simultaneously. The resultant yeast transformants expressing both vectors did not grow on the SD−Leu−Trp−His medium, which is indicative of the impossibility of Lws1 to possess SDR enzyme activity. This preliminary observation is consistent with the previously reported SDR protein 17β-HSDcl, which also did not exhibit the capacity to form protein aggregates (Figure 3B).

### 2.4. Expression of Lws1 under Different Nutrition Conditions in L. theobromae

**Figure 4:**
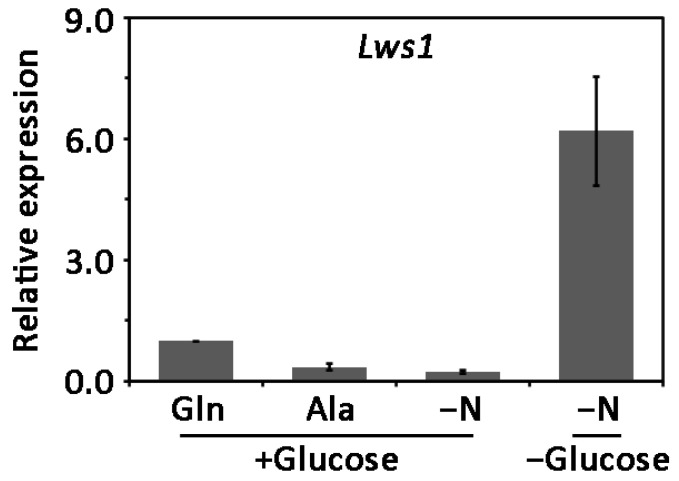
Transcription profiling of *Lws1* under different nutrition conditions. The vegetative hyphae of the wild type were cultured in liquid minimal media for 36 h and then transformed into minimal media supplemented with different nutrition metabolites at a final concentration of 10 mM for an additional 4 h. The total RNA of the cultured vegetative hyphae was isolated and then reversely transcribed into cDNA for gene expression analyses. Relative transcript levels of *Lws1* were calculated using the 2^−ΔΔCT^ method. Relative transcript levels of *Lws1* under different nutrition conditions were normalized by the *actin* gene and calibrated against that of the minimal medium added with glutamine. For the minimal medium, 1% glucose was used as the sole carbon source and 10 mM glutamine (Gln) or alanine (Ala) was the sole nitrogen source. Standard errors were derived from three independent experiments.

To examine whether *Lws1* was involved in nutrition metabolism regulation, we attempted to detect the transcript accumulation of *Lws1* with cDNA reversely transcribed from RNA isolated from vegetative hyphae cultured with different nitrogen and carbon sources. Statistical data showed that, compared to the nitrogen-sufficient condition (Gln), the expression level of *Lws1* was obviously reduced under nitrogen-limiting (Ala) and nitrogen-starvation (−N) states. Moreover, the decreased expression of *Lws1* under the nitrogen-starvation condition (−N) was prevented by the simultaneous starvation for a carbon source (−N−C) (Figure 4). Altogether, these results suggest that Lws1 responds to external nitrogen and carbon sources and may mediate nutrition metabolism in *L. theobromae*.

## 3. Discussion

The nitrogen and carbon metabolism regulations have been widely investigated in various model microorganisms, including *A. nidulans* [2,3,5], *N. crassa* [7,9], *M. grisea* [14], *F. graminearum* [18], and *S. cerevisiae* [11,12,13], but far less has been characterized in the opportunistic plant pathogen *L. theobromae*. In the current study, we set out to investigate the nitrogen and carbon metabolism used by *L. theobromae* to grow and colonize inside grapevine tissues. We focused on whether nitrogen and carbon metabolism regulators were involved in disease development caused by *L. theobromae*. Our results suggest that *L. theobromae* deploys two transcription regulators, Lws1 and LtAreA1, to mediate fungal virulence and nutrition metabolism. Lws1 has a negative impact on visible lesion growth, which differs from the roles most virulence factors serve. However, it has been found that NmrA-like proteins also negatively mediate the pathological processes in other pathogens. The *PcNMRAL1* overexpression line in *Phytophthora capsici* displayed a reduction in colony expansion, pathogen biomass, and disease lesions in comparison with the wild type [45]. Additionally, the overexpression of *PcNmrAL1* also prolonged the expression of the biotrophy marker gene *PcHmp1I,* indicating that the *PcNmrAL1* overexpression line has a prolonged biotrophic phase, which is coupled with reduced lesion development [45]. Moreover, deletion of *nmrA* in *A. flavus* resulted in increased conidiation and sclerotia production on glucose minimal medium (GMM) supplemented with ammonium [17]. Consistent with its homologs in other fungi, *Lws1* was transcriptionally reduced under nitrogen-insufficient or nitrogen-starvation conditions, compared to nitrogen-sufficient conditions. Additionally, decreased transcription under nitrogen-starvation conditions was prevented by the simultaneous starvation for carbon sources, suggesting that Lws1 may be involved in both nitrogen and carbon source metabolism in *L. theobromae*. Considering these similar results, it can be assumed that, to some extent, the molecular role of the NmrA protein is conservative among these species, and the NmrA protein may play a major role during the biotrophic phase for nutrition acquisition and the maintenance of individual life. It is also possible that the existence of NmrA proteins in microorganisms may be a result of horizontal gene transfer from hosts, which is conducive for hosts to defeat pathogens via NmrA regulation. Moreover, seed infection assays on living peanut cotyledons revealed that NmrA is required for the invasive virulence of *A. flavus*, which is consistent with its reduced conidia production on peanut cotyledons [17]. When *A. flavus* was germinated under different conditions (host seeds and nutrition-sufficient media), the NmrA protein exerted opposite influences on fungal conidiation, suggesting that the molecular roles of NmrA in pathological development were also affected by external nitrogen states.

Previous reports supported that the regulation of AreA activity involved the NmrA protein interacting with the DNA-binding domain and extreme C-terminus of AreA to prevent the activation of nitrogen catabolic genes [3,25,26]. Unlike previous results, we verified that the LtNmrA protein interacted with the LtAreA protein via the N-terminal fragment, rather than the DNA-binding domain and extreme C-terminus. This difference may arise from the function redundancy of NmrA proteins, as many NmrA-like proteins have been predicted based on previous genome assembly and function annotation in *L. theobromae*. The molecular roles of some unidentified NmrA-like proteins in *L. theobromae* may more closely resemble those of NmrA in *A. nidulans* [3,25,26] and NMR1 in *N. crassa* [46]. Therefore, more attention should be paid to NmrA-like proteins in further research, especially in the latent pathogen *L. theobromae*, which appeared to secrete more degradative enzymes to degrade host tissues for nutrition acquisition during our previous work [37].

It has been found that proteins belonging to the SDR superfamily usually function as dimers or tetramers, and dimerization is a prerequisite for their enzyme activity [27,31]. Here, we did not detect a protein–protein interaction between Lws1 monomers, which was also featured by another two SDR proteins, 17β-HSDc of *Cochliobolus lunatus* [31] and NmrA of *A. nidulans* [27,28], suggesting that NmrA proteins in fungi may serve as transcription regulators rather than catalysis enzymes.

In filamentous fungi, such as *A. nidulans* and *N. crassa*, the AreA homolog was annotated as a GATA family transcription activator with a critical C-terminal motif [7,19]. Mutation analyses revealed that the extreme C-terminus of the AreA protein in *A. nidulans* is sufficient for the appropriate modulation of AreA function [19] (Platt et al., 1996). Structural comparison showed that the C-terminal motif is highly conserved in the homologs from *A. nidulans* (AreA), *N. crassa* (NIT2), and *L. theobromae* (LtAreA), implying similar structures and conserved functions of their C-termini. However, we cannot determine the molecular roles of the short motif in *L. theobromae* due to the multinuclear structure of this fungus. This technically constrains us to engineer deletion mutants for the targeted gene. Further breakthroughs in the genetic transformation of *L. theobromae* will be conducive for investigations of the molecular roles of pathogenicity-related factors in this fungus.

In conclusion, we demonstrated that the Lws1 protein negatively regulated disease development during infection. Similar to its homologs in other fungi, Lws1 responded to external carbon and nitrogen nutrition. Moreover, the Lws1 protein interacted with the GATA transcription factor LtAreA. These findings shed light on the nutrition metabolism and pathogenesis of *L. theobromae*.

## 4. Materials and Methods

### 4.1. Fungal and Bacterial Strains, Growth Conditions, and Plant Material

The *L. theobromae* strains including the wild type, overexpressed transformants, and silenced transformants were cultured on complete medium (0.6% yeast extract, 0.3% casein acid hydrolysate, 0.3% casein enzymatic hydrolysate, and 1% sucrose) at 28◦C. The *Escherichia coli* (*E. coli*) strain BL21 was cultured in Luria–Bertani (LB) medium (0.5% yeast extract, 1% g tryptone, 1% g NaCl per liter, and 1.6% agar for plates). Healthy green shoots of *Vitis vinifera* cv. ‘Summer Black’ were collected from the Xiangyi vineyard in Beijing. Ampicillin was added to the LB medium at a final concentration of 50 µg/mL.

### 4.2. RNA Extraction and Real-Time Quantitative PCR (qRT-PCR)

Vegetative hyphae cultured under different nutrition conditions were harvested for RNA extraction. Total RNA was extracted using TRIzol reagent (Invitrogen, Carlsbad, CA, USA) and then reversely transcribed into cDNA with a TransScript^®^ One-Step gDNA Removal and cDNA Synthesis SuperMix kit (TransGen Biotech, Beijing, China). The qRT-PCR was performed in an ABI 7500 Real-Time system (Applied Biosystems, Waltham, MA, USA) and conducted in 20 µL volumes composed of 10 µL RealStar Green Fast Mixture with ROX II (GenStar Biosolutions, Beijing, China), 1.0 µL cDNA, 0.2 µM primer, and 8.2 µL sterile ddH_2_O. The PCR program progressed as follows: denaturation at 95 °C for 2 min, followed by 40 cycles of 95 °C for 15 s, and 60 °C for 30 s. The *actin* gene was used as the internal control. Relative expression of the target gene was calculated using the 2^−ΔΔCT^ method [47]. All the experiments were replicated at least thrice independently with three repeats each. All the primers used in the study are listed in Table S1.

### 4.3. Pathogenicity Tests of Overexpressed and Silenced Transformants of the Lws1 Gene

For overexpression assays, the open reading frame (ORF) of *Lws1* was amplified with the primer pair NmrA1OE-f/NmrA1OE-r (Table S1) and then subcloned into the *PtrpC* promoter-driven vector *pKSNTP*. Next, the fusion construct, named *pKSNTP-Lws1*, was transformed into *L. theobromae* protoplasts using the polyethylene glycol (PEG)-mediated transformation method [42]. Resultant transformants were screened against neomycin resistance and further confirmed by qRT-PCR analyses. Two positive transformants, referred to as Lws1-OE1 and Lws1-OE2, were selected for pathogenicity tests on detached green shoots of the susceptible *V. vinifera* cv. ‘Summer Black’. The wounds on the grapevine shoots were inoculated with the mycelial plugs of the wild-type, Lws1-OE1, and Lws1-OE2 strains and then maintained in a growth chamber with constant humidity and temperature. The lesion length of the infected grapevine shoot was measured at 3 days post-inoculation (dpi). At least five biological repeats of each *Lws1*-overexpressed transformant were performed.

For gene silencing, we amplified the sense and antisense fragments with the primer pairs Lws1RNAi-Sf/NmrA1RNAi-Sr and Lws1RNAi-ASf/NmrA1RNAi-ASr, respectively. Subsequently, both fragments were ligated into the *pRNT* vector in the given order. The fusion vector was transformed into *L. theobromae* protoplasts. The protocols used for the transformation and pathogenicity tests of silenced transformants were similar to those used for the overexpressed transformants.

### 4.4. Yeast Two-Hybrid Assay

The ORF of *Lws1* was amplified with the primer pairs shown in Table S1 and then cloned into the *pGBKT7* vector as the bait vector. Similarly, the ORF of *LtAreA* was amplified with the primer pairs listed in Table S1 and then ligated into the *pGADT7* vector as the prey vector. Subsequently, both the bait and prey vectors were co-transformed into yeast AH109, using the lithium acetate method described by Becker and Lundblad [48]. Resultant transformants were tested for their growth on a synthetic dropout (SD-Leu-Trp-His) medium.

## Data Availability

The data that support the findings of this study are available in all figures and tables of this article.

## Supplementary Materials

The following supporting information can be downloaded at: https://www.mdpi.com/article/10.3390/plants11172197/s1, Table S1: Primers used in this study.
