# Supplementary material for: An NmrA-Like Protein, Lws1, Is Important for Pathogenesis in the Woody Plant Pathogen Lasiodiplodia theobromae
Source: Plants (Basel). 2022 Aug 24;11(17):2197. doi: 10.3390/plants11172197 (PMC9460803; doi:10.3390/plants11172197)
Supplement: Supplementary file 1 [file plants-11-02197-s001.zip › Table S1.pdf]

Table S1 Primers used in this study.

Primer name	Sequence （5’-3’）	Use of primer
Lws1$^{1-246}$B-f	GCCATGGAGGCCGAATTCATGGCCAAGCAGCGTGTTCT	Used for the construction of *pGBKT7-Lws1*$^{1-246}$ vector
Lws1$^{1-246}$B-r	ACGGATCCCCGGGAATTCTTACTCCTTTACTTTCGCCTC	
Lws1$^{247-324}$B-f	GCCATGGAGGCCGAATTCATGGCCAAGATTAACGGCGA	Used for the construction of *pGBKT7-Lws1*$^{247-324}$ vector
Lws1$^{247-324}$B-r	ACGGATCCCCGGGAATTCTCACAAGAAGTCGTAGTGC	
Lws1B-f	GCCATGGAGGCCGAATTCATGGCCAAGCAGCGTGTTCT	Used for the construction of *pGBKT7-Lws1* vector
Lws1B-r	ACGGATCCCCGGGAATTCTCACAAGAAGTCGTAGTGC	
Lws1A-f	GCCATGGAGGCCAGTGAATTCATGGCCAAGCAGCGTGTT	Used for the construction of *pGADT7-Lws1* vector
Lws1A-r	CAGCTCGAGCTCGATGGATCCTCACAAGAAGTCGTAGTGC	
AreA$^{1-480}$A-f	ATGGAGGCCAGTGAATTCATGTCGGACGCGCAACA	Used for the construction of *pGADT7-LtAreA*$^{1-480}$ vector
AreA$^{1-480}$A-r	CCCACCCGGGTGGAATTCTCATCCCGCTGCCGAAACGTTG	
AreA$^{481-929}$A-f	ATGGAGGCCAGTGAATTCATGCCACCTCCGCCTTTCTCG	Used for the construction of *pGADT7-LtAreA*$^{481-}$

AreA$^{481-929}$A-r	CCCACCCGGGTGGAATTCCTACAAGCTCATTGTCAGC	^{929} vector
AreA$^{1-121}$A-f	ATGGAGGCCAGTGAATTCATGTCGGACGCGCAACA	Used for the construction of *pGADT7-LtAreA*$^{1-121}$ vector
AreA$^{1-121}$A-r	CCCACCCGGGTGGAATTCTCATTGGGTGCCTATGGGGT	
AreA$^{122-149}$A-f	ATGGAGGCCAGTGAATTCATGATATGGAAGCTTTACAACAA	Used for the construction of *pGADT7-LtAreA*$^{122-149}$ vector
AreA$^{122-149}$A-r	CCCACCCGGGTGGAATTCTCACATCGACATCATCCGCC	
AreA1$^{150-480}$A-f	ATGGAGGCCAGTGAATTCATGAACCTGCGGAAAATGCGTA	Used for the construction of *pGADT7-LtAre*$^{150-480}$ vector
AreA1$^{150-480}$A-r	CCCACCCGGGTGGAATTCTCATCCCGCTGCCGAAACGTTG	
Lws1OE-f	ATGAATTCATGGCCAAGCAGCGTGTTCT	Used for the construction of *Lws1* overexpression vector
Lws1OE-r	CATGGATCCTCACAAGAAGTCGTAGTGC	
Lws1RNAi-Sf	ATAAGCTTGGAGCAAGTGTACAATC	Used for the construction of *Lws1* RNAi vector
Lws1RNAi-Sr	TATGAATTCCATCGCTGCACGTGAA	
Lws1RNAi-ASf	AACTGCAGTGATGTTTGGCCGCT	
Lws1RNAi-ASr	ATAGGATCCGGAGCAAGTGTACAA	
